# Laparoscopic cervico-isthmic anastomosis for old traumatic disjunction between the cervix and the uterine corpus: a case report and literature review

**DOI:** 10.1186/s12905-023-02263-w

**Published:** 2023-03-15

**Authors:** Hongxia Zhu, Minjiao Zhu, Jun Wang

**Affiliations:** Department of Gynecology, The International Peace Maternal and Child Health Hospital, School of Medicine, Shanghai Jiao Tong University, 910 Hengshan Road, Xuhui District, Shanghai, 200030 China

**Keywords:** Laparoscopy, Cervico-isthmic disjunction, Cervico-isthmic anastomosis, Pelvic trauma, Primary amenorrhea, Cyclic abdominal pain

## Abstract

**Background:**

Old traumatic disjunction between the cervix and the uterine corpus is very rare case. In most cases, it is not immediately noticed until the onset of other symptoms, such as amenorrhea, periodic abdominal pain and so on. Scanty cases of anastomosis surgery via laparoscope have been reported.

**Case presentation:**

We report here a 23-year-old young woman with the primary amenorrhea due to traumatic cervico-isthmic disjunction. The patient had a closed pelvic fracture at the age of 4 and has experienced periodic lower abdominal pain since the age of 17 years. A complete disjunction between the cervix and the uterine corpus was diagnosed. Laparoscopic cervico-isthmic anastomosis was performed to restore the continuity of the endometrial cavity and cervical canal. After this surgery, normal menstruation was resumed without cyclic abdominal pain.

**Conclusion:**

Laparoscopic cervico-isthmic anastomosis could reconstruct the uterine outflow tract successfully, alleviate symptoms, and achieve a good short-term outcome.

## Background

Cervico-isthmic disjunction has two types, congenital dysplasia and traumatic. Both types are extremely rare. The former is mostly accompanied by other genitourinary developmental abnormalities, while the latter is mostly caused by pelvic trauma and might be combined with the other damage on the genital organs. Traumatic disjunction between the cervix and the uterine corpus is usually unnoticed in childhood due to the absence of symptoms. While growing up, patients have the secondary sexual characteristics in normal level. As a consequence of the uterine outflow tract obstruction, in adolescence the related clinical symptoms may appear, such as amenorrhea, periodic abdominal pain, endometriosis, adenomyosis or even infertility, etc.

## Case presentation

**Fig. 1:**
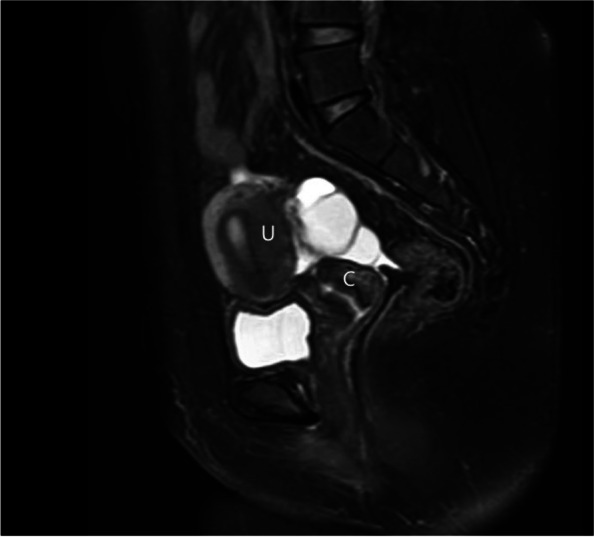
MRI examination showed a complete separation of the cervix (C) from the uterine corpus (U)

A 23-year-old nulligravida with a history of primary amenorrhea was referred to our hospital for surgical treatment. Her main complaint was that since the age 17 she has had the cyclic lower abdominal pain for 2–3 days once a month. She had normal breast development at age of 13 and never had cyclical menstrual symptom. She reported a history of a pelvic trauma during a vehicle accident at age of 4, which was handled nonsurgically. When consulting in another hospital in Shanghai in July 2021, her pelvic magnetic resonance imaging (MRI) suggested a complete disjunction between the cervix and the uterine corpus combined with the bilateral adnexal cysts (Fig. 1). In our hospital, the physical examination suggested that the normal secondary sexual characteristics were well present, with normal the vulva and vagina etc. Furthermore, it revealed that the cervix was mobile while the uterine corpus was relatively fixed. Ultrasonographic examination suspected a disjunction between the cervix and uterine corpus, meantime detected the bilateral adnexal cysts as 4 cm in left and 6 cm in right. There was no indication of abnormal urinary system in intravenous pyelography (IVP). Serum endocrine examination was normal as expected, with FSH:6.60IU/L, LH:2.64IU/L, E2 117.28pmol/L, P:3.41nmol/L, PRL:219.37uIU/ml, T: 2.39 nmol/L, AMH:3.9ng/ml.

**Fig. 2:**
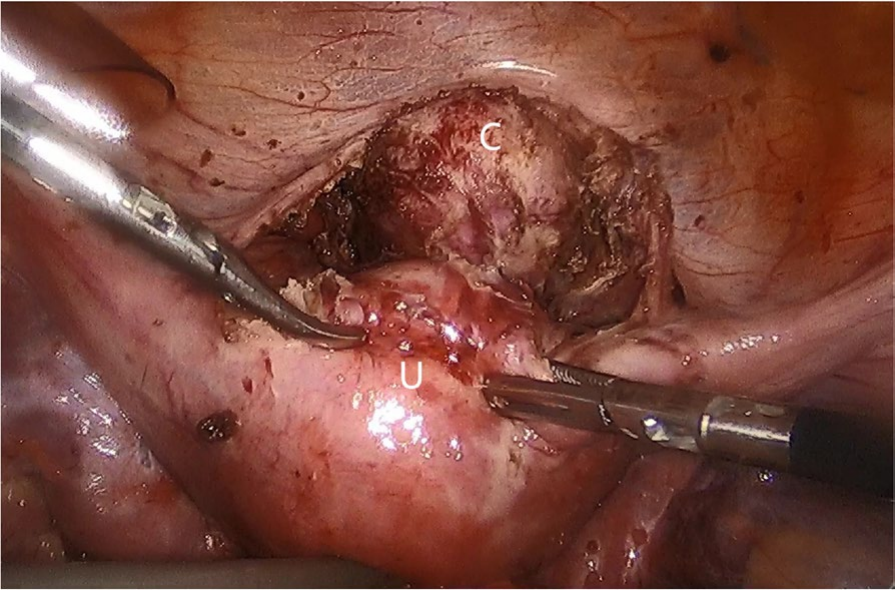
At laparoscopy the uterine isthmus was completely separated from the cervix (C), and the uterine corpus (U) was connected to the cervix by the broad ligament

**Fig. 3:**
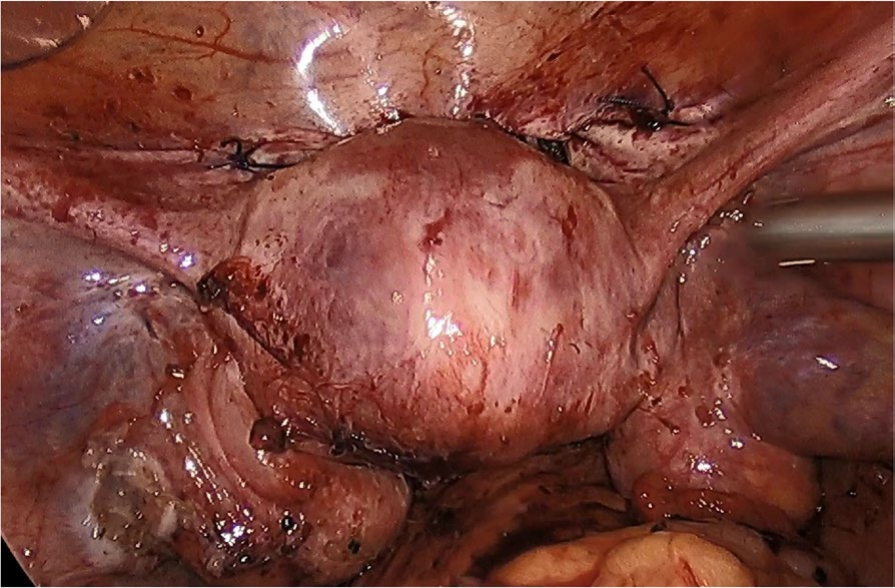
Anterior image of the uterine after anastomosis

Given the trauma history and imaging assessment, old traumatic disjunction between the cervix and the uterine corpus was considered as diagnosis. The laparoscopic exploration under general anesthesia confirmed the complete separation of cervix from the uterine corpus (Fig. 2). The extensive adhesions, scattered peritoneal endometriotic lesions in the pelvis and bilateral endometrioma were detected. Laparoscopic surgery separated the pelvic adhesions and removed the endometrial cysts. The bilateral broad ligaments were detached from the severed isthmus with 3 cm apart. The vesicouterine space was dissected with 3 cm apart below the blind end of the cervix. Hegar’s dilatators dilated the cervical canal via vaginal gradually with different size number of rod. At the end, the No. 10 dilated rod was inserted as a guide for the inner cervical orifice. The upper blind end of the cervix was dissected down to the cervical canal. Pituitrin 3u diluted in 5ml saline was injected into the myometrium, which resulted in the contraction. Hence, it obviously appeared the convex weak end of the corpus, which assisted to identify the incision site. The protruding site was dissected until the uterine cavity. An end-to-end anastomosis between the cervix and the uterine corpus was performed circumferentially by using 1 − 0 absorbable knotless suture. Continuous suturing was done between the posterior part of the corpus and the cervix, then for the anterior plane. The serosal layer of the isthmus was sutured for reinforcement (Fig. 3). A Foley catheter with 3ml balloon was inserted into the uterine cavity through the newly formed canal, in order to prevent adhesion and stenosis of the anastomotic site. The surgery took in total 180 min with the approximate 100 ml blood loss.

**Fig. 4:**
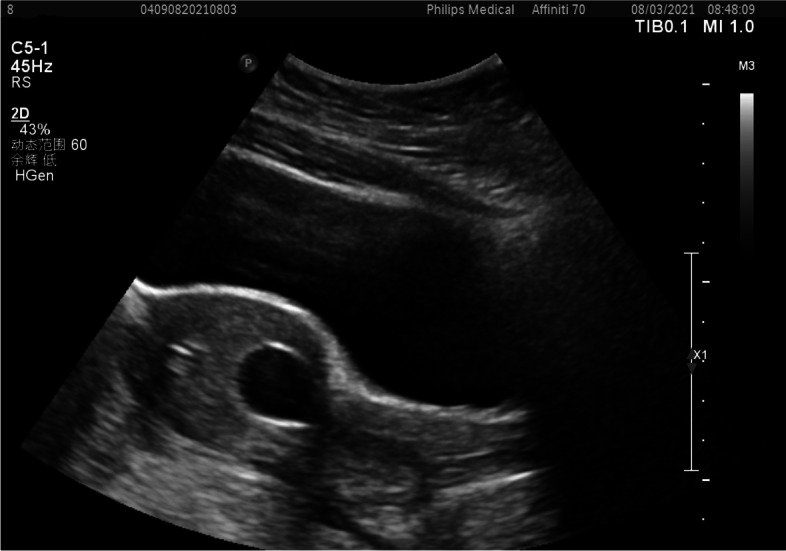
Ultrasonographic examination showed that Foley catheter had been placed correctly with the balloon inflated in the lower segment of the uterine cavity and myometrium of the uterine isthmus was continuous

On day 4 after operation, transabdominal ultrasound examination indicated that Foley catheter together with the inflated balloon located as expected in the lower portion of the uterine cavity and myometrium of the uterine isthmus was continuous (Fig. 4). The Foley catheter was removed day 6 after operation and the patient was discharged on the next day. Additionally, endometriosis lesions were removed, and further confirmed as endometriosis by histology.

**Fig. 5:**
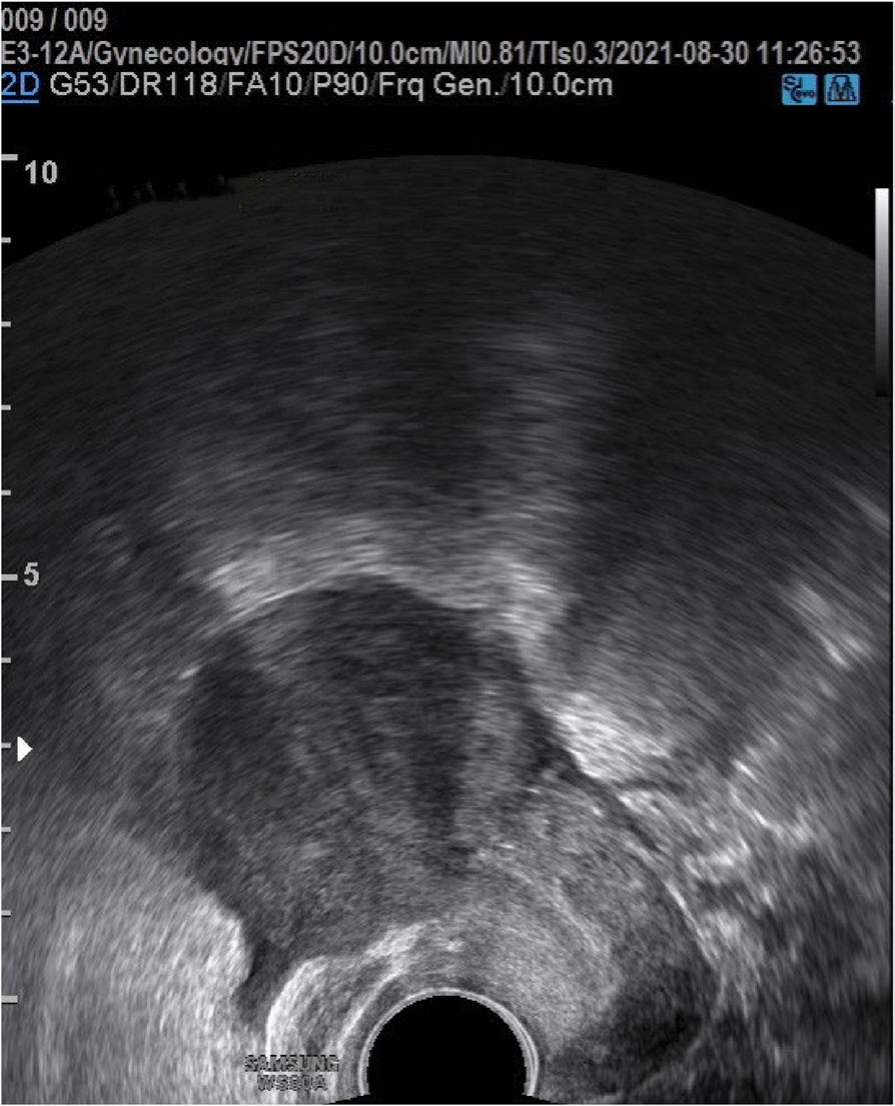
The ultrasound examination at one month after surgery showed the myometrium of the lower uterine segment was continuous

**Fig. 6:**
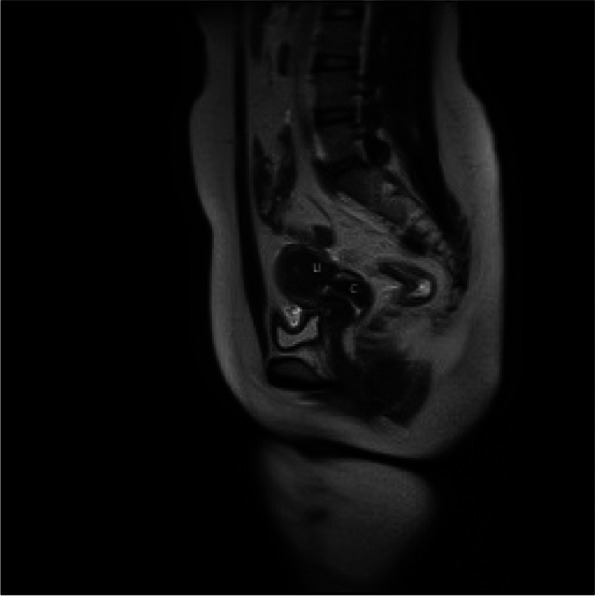
MRI examination three months after surgery showed a normal uterus (U) and cervix (C) with the myometrial continuity of the isthmus

Menses occurred 23 days postoperatively without pelvic pain. On the fifth day of menstruation, a sequential use of Estrogen and progestin (estradiol valerate at a dose of 4 mg daily for 21 days with continued addition of 10 mg progesterone for the last 10 of the 21 days) was initiated, in order to accelerate the wound healing in uterine and completely restore the function of the uterus. After three artificial menstrual cycles, combined oral contraceptives were used to prevent endometriosis recurrence. At one month after surgery, the ultrasound examination showed that the myometrium of the lower uterine segment was continuous (Fig. 5) and there was no distinct mass in bilateral adnexal areas. MRI examination three months after surgery showed a normal uterus and cervix with the myometrial continuity of the isthmus (Fig. 6). At the 8-month postoperative follow-up visit, the patient has regular, normal menstrual flow without low abdominal pain and substantial dysmenorrhea.

## Discussion and conclusions

Cervico-isthmic disjunction is extremely rare condition. The patient with a normal development of the cervix and the corpus but a transection isthmus, indicated that this transection occurred after the fusion and lumenization of the bilateral müllerian ducts had completed. Thus these findings were not consistent with müllerian hypoplasia. Given the history of pelvic fracture in childhood, old traumatic disjunction between the cervix and the uterine corpus was suspected.

The female reproductive organs are protected by the pelvic bony structure and rarely subjected to injuries. Pelvic fracture, a hallmark of higher energy trauma, may deform the pelvis and damage the pelvic organs. Up to date, only six similar cases were reported ( [1–6], Table 1). All the patients had history of pelvic fracture in childhood caused by serious accidents. But their cervico-isthmic disjunction was only detected several years later as juvenile females. The cervix is longer, which is 2:1 ratio to the length of uterine corpus. The cervix is relatively fixed to the pelvic sidewall by the cardinal ligaments, while the uterine corpus is hold by round ligaments and broad ligaments is relatively mobile. When a high-energy trauma occurs, the opposite tractions force on the cervix and the corpus generate shear force on the isthmus, which results in the complete fracture of the isthmus as the slenderest part of the uterus. The uterine rupture can be detected immediately in an emergency surgery, in the case of active bleeding or other symptoms due to pelvic fracture [7, 8]. Some cases of cervico-isthmic disjunction are not immediately noticed until the onset of other symptoms, such as amenorrhea, periodic abdominal pain, infertility, ovarian endometriomas, and so on.

**Table 1:** Published cases of reparation for old traumatic disjunction between the cervix and the uterine corpus

Publication and year	Age at pelvic trauma (years)	Age at presentation (years)	Symptom	Surgery	Pregnancy
Murphy 1993 [1]	2	22	primary amenorrhea	Laparotomy	3 children by C-section
Donner 2000 [2]	12	18	primary amenorrhea cyclical abdominal discomfort	Laparotomy	IVF aged 32Y, 1 child by C-section
Kesterson 2007 [3]	13	15	secondary amenorrhea cyclical abdominal pain	Laparotomy	No pregnancy desired yet
Rashmi 2009 [4]	3	15	primary amenorrhea cyclical abdominal pain	Laparotomy	No pregnancy desired yet
Zhang 2013 [5]	2	16	primary amenorrhea cyclical abdominal pain	Laparoscopy	No pregnancy desired yet
Vignolle 2018 [6]	4	36	Primary infertility	Laparotomy	No pregnancy yet

*IVF* In Vitro Fertilization

Medical history and the following examinations are helpful in making the diagnosis. Detailed medical history is required, especially about trauma such as pelvic fracture. The secondary sexual characteristics were well developed. On gynecological examination, the vulva, the vagina and the cervix appear normal as well as a normal or enlarged uterine corpus. The findings might include the presence of an adnexal mass that most likely is an ovarian endometrioma. Ultrasound is the preferred detection method to directly visualize the cervix and uterine cavity morphology, the presence of pelvic endometriotic cysts and intrauterine or pelvic fluid accumulation. MRI can provide excellent anatomic detail and soft-tissue contrast, which is important for preoperative evaluation and differential diagnosis. Intravenous pyelography (IVP) or CT urography (CTU) were performed to check the urinary system for the presence of any abnormalities. Tumor markers are mostly within the normal range, and blood CA125 levels may be elevated in the presence of endometriosis. The Chromosomal examination was normal. Female sex hormone levels are mostly within the normal range.

Traumatic disjunction between the cervix and the uterine corpus needs to be differentiated from complete obstructive genital malformations, such as congenital cervical atresia, transverse vaginal septum, imperforate hymen, etc. Detailed clinical history and imaging modalities such as ultrasonography or MRI are helpful for diagnosis and differential diagnosis.

Once cervico-isthmic disjunction is confirmed, it should be treated surgically as soon as possible. The aim of surgery is first to relieve symptoms and second to preserve fertility function. Compared with trauma, the delay in surgery does not affect the likelihood of anatomical recovery, however, early diagnosis can avoid severe pelvic endometriosis and infertility. Among patients with congenital cervical atresia, early diagnosis and surgery seem to be necessary to prevent endometriosis [9]. Evidence of pelvic endometriosis(including peritoneal endometriosis, ovarian endometriosis, deep infiltrating endometriosis)and/or infertility were reported in most published cases of old traumatic disjunction between the cervix and the uterine corpus [2, 4–6]. Old traumatic disjunction between the cervix and the uterine corpus mostly occurs in young women, and fertility preservation is particularly important for them. Uterocervical anastomoses have shown their effectiveness in restoring anatomy and function, therefore, efforts should be made to preserve uterine and fertility function for the patient if the conditions permit. In previous literature reports, There were two surgical approach for the reconstructive surgery, laparotomy and laparoscopy. Compared with laparotomy, laparoscopy has advantages including cosmetic incisions, less trauma, less postoperative pain, fewer complications and rapid recovery. And for those with mature experience in laparoscopic surgery, laparoscopy has the advantages of a clear and accurately magnified surgical field to facilitate the achievement. There are also some key points during laparoscopic surgery, which facilitate the operation: Firstly, the timing of surgery is recommended during menstruation [10], when the patient’s periodic abdominal pain occurs, for the most hematochezia in the uterine cavity helps intraoperatively judge the incision site of the uterine corpus. Our patient was already in the early secretory phase when she underwent surgery at our institution, it is difficult for us to find the broken end of the uterine corpus. Intraoperatively, we injected pituitrin into the uterine myometrium to contract it, and the weak proximal uterine cavity of the broken end can be obviously convex, which helped us to find the incision site of the corpus. Secondly, Hegar’s dilatator was inserted into the cervical canal as a guide via the transvaginal route, which made it less difficult for us to find the inner cervical orifice. Thirdly, laparoscopic suturing is the most difficult task in the surgery. We used absorbable knotless suture and continuous suturing was done between the posterior part of the corpus and the cervix, then for the anterior plane. The serosal layer of the isthmus was sutured for reinforcement.

Postoperatively, prompt management is warranted to prevent adhesion of the anastomotic site and cervical stent seems to be a good choice. In patients with congenital cervical atresia, the cervical canal with no columnar epithelium but fibrous connective tissue is highly prone to adhesion, stenosis, and atresia postoperatively. It has been reported that 60% patients with a preserved uterus underwent a second surgery because of re-obstruction of the neocervical canal in patients with congenital cervical atresia [9]. Our case was a traumatic cervico-isthmic disjunction with a well-developed mucosa of the cervical canal, we still need to take measures to prevent adhesion of the anastomotic site. There is no standard duration of cervical stent placement and the placement duration in previous reports ranged from 10 days [5, 6] to more than 2 months [3]. In our case, we use a Foley catheter inserted into the uterine cavity through the newly formed canal, usually with an intra-balloon infusion ≤ 5 ml and left in place for 5–7 days. And sufficient anti-infection treatment is recommended for reducing the intraoperative risks and postoperative occurrence of infection. The endometrium was chronically compressed by repeated hematometra. After cervico-isthmic anastomosis, some measures were needed to promote endometrial regeneration and coverage of the anastomotic site. Hormonal therapy represented valid option to regenerate the endometrium [11]. Experience in frozen embryo transfer, demonstrated that the hormonal endometrium preparation using sequential treatment with oestrogen and progesterone, is effective in endometrial growth also by excluding the ovarian functionality [12]. As postoperative treatment, two to three artificial menstrual cycles were induced to promote endometrial repair and reduce the risk of postoperative cervico-isthmic adhesion and stenosis.

In patients who are not currently planning to conceive, medical therapy is usually administered after surgery to reduce the rate of postoperative cyst recurrence and pain recurrence [13]. Most published guidelines [13, 14] recommend using a combined oral contraceptive (COC) as the first line of hormonal treatment for endometriosis. The recent systematic reviews [15, 16] have demonstrated that the postoperative use of COCs reduced the risk of disease recurrence, particularly when administered in the long term. Our patient began to take COCs to prevent endometriosis recurrence after 3 months of surgery. In patients experiencing postoperative infertility, tubal incompetence and diminished ovarian reserve, assisted reproductive technology is needed if necessary [14]. In patients with successful pregnancies, some researchers think that the obstetric risk of such procedure is gestational or intrapartum uterine rupture with a risk comparable to that of cesarean section or evacuation of intramural fibroids [6]. Termination of pregnancy by cesarean section at 37–39 weeks of gestation has been considered acceptable [6].

In summary, amenorrhea with cyclic lower abdominal pain in patient with pelvic traumatic history should be on the alert for old traumatic disjunction between the cervix and the uterine corpus. It is not so difficult to make the diagnosis from the time it was evoked. Laparoscopic cervico-isthmic anastomosis is a minimally invasive way to reconstruct the uterine outflow tract successfully, alleviate symptoms, and obtain better short-term outcomes. Further case studies and longer follow-up are needed to better understand whether laparoscopic approach has advantages over laparotomy in the recovery of postoperative fertility.

## Data Availability

All data generated or analysed during this study are included in this published article.

## Abbreviations

MRI: magnetic resonance imaging
IVP: intravenous pyelography
